# A Digital Approach for Addressing Suicidal Ideation and Behaviors in Youth Mental Health Services: Observational Study

**DOI:** 10.2196/60879

**Published:** 2024-12-18

**Authors:** Min K Chong, Ian B Hickie, Antonia Ottavio, David Rogers, Gina Dimitropoulos, Haley M LaMonica, Luke J Borgnolo, Sarah McKenna, Elizabeth M Scott, Frank Iorfino

**Affiliations:** 1 Brain and Mind Centre The University of Sydney Camperdown Australia; 2 headspace Bondi Junction Sydney Australia; 3 headspace Port Macquarie Youth Services Port Macquarie Australia; 4 Faculty of Social Work University of Calgary Calgary, AB Canada; 5 School of Medicine University of Notre Dame Sydney Australia

**Keywords:** mental health service, youth mental health, suicide management, clinical decision support, primary care, personalization, suicide, suicidal, youth, mental health, mental health care, suicide prevention, digital technology, online assessment, clinician, digital health, health informatics, clinical information

## Abstract

**Background:**

Long wait times for mental health treatments may cause delays in early detection and management of suicidal ideation and behaviors, which are crucial for effective mental health care and suicide prevention. The use of digital technology is a potential solution for prompt identification of youth with high suicidality.

**Objective:**

The primary aim of this study was to evaluate the use of a digital suicidality notification system designed to detect and respond to suicidal needs in youth mental health services. Second, the study aimed to characterize young people at different levels of suicidal ideation and behaviors.

**Methods:**

Young people aged between 16 and 25 years completed multidimensional assessments using a digital platform, collecting demographic, clinical, social, functional, and suicidality information. When the suicidality score exceeded a predetermined threshold, established based on clinical expertise and service policies, a rule-based algorithm configured within the platform immediately generated an alert for treating clinicians. Subsequent clinical actions and response times were analyzed.

**Results:**

A total of 2021 individuals participated, of whom 266 (11%) triggered one or more high suicidal ideation and behaviors notification. Of the 292 notifications generated, 76% (222/292) were resolved, with a median response time of 1.9 (range 0-50.8) days. Clinical actions initiated to address suicidality included creating safety plans (60%, 134/222), conducting safety checks (18%, 39/222), psychological therapy (8%, 17/222), transfer to another service (3%, 8/222), and scheduling of new appointments (2%, 4/222). Young people with high levels of suicidality were more likely to present with more severe and comorbid symptoms, including low engagement in work or education, heterogenous psychopathology, substance misuse, and recurrent illness.

**Conclusions:**

The digital suicidality notification system facilitated prompt clinical actions by alerting clinicians to high levels of suicidal ideation and behaviors detected among youth. Further, the multidimensional assessment revealed complex and comorbid symptoms exhibited in youth with high suicidality. By expediting and personalizing care for those displaying elevated suicidality, the digital notification system can play a pivotal role in preventing rapid symptom progression and its detrimental impacts on young people’s mental health.

## Introduction

The high prevalence of suicidal ideation and behaviors among youth is alarming [1]. While the biological and psychosocial factors associated with the development of suicidality are complex [2] and alone cannot predict the risk of suicide, it is strongly associated with long-term severe mental illnesses [3], poor social functioning, [1], and future suicidal behaviors [4,5]. Therefore, effective identification and management of suicidality in youth mental health services is crucial for detecting individuals with complex needs and preventing further progression of symptoms [6,7].

Conducting a thorough and efficient assessment of suicidality poses time and labor constraints on services with high demand [8]. Such barriers often lead to delays in screening, timely responses, and the delivery of appropriate interventions. Extended wait times can be especially unsafe for young people experiencing intense levels of suicidal ideation and behaviors [9,10].

Digital technology is a potential solution for operationalizing timely assessments in mental health services [7]. Further, a digital notification system that can alert severe or sudden changes in a client’s mental health symptoms can facilitate prompt care coordination and allocation. The clinical use and feasibility of such systems have been generally well-accepted in other areas of medicine [11-13].

In 2017, a digital notification system that detects suicidal ideation and behaviors in youth mental health services was developed and evaluated [14]. This pilot study demonstrated its effectiveness for triaging young people with high levels of suicidality into appropriate care in large general practitioner practices and youth mental health services [14,15]. However, the longitudinal feasibility of this system has not yet been explored.

Therefore, this study aims to evaluate the use of a digital suicidality notification system in youth mental health services over a 5-year period in a naturalistic setting. The current study reports the rate and type of clinical responses initiated by suicidality notifications and identifies the clinical, social, and functional needs of young people at different levels of suicidality.

## Methods

### Recruitment

Young people aged between 16 and 25 years, who presented to primary (headspace [16]) and community mental health services between November 2018 and October 2023 were invited to participate. All young people who were within the age range and used a digital platform, Innowell (University of Sydney and PwC), were eligible to participate in this study.

### Participant Procedures

All participants filled out self-reported, multidimensional assessments before their initial consultation as part of the intake process at participating services. After the mandatory initial assessment, reassessment was encouraged for symptom monitoring throughout care. The questionnaire was administered through the Innowell platform, a web-based platform that supports individuals’ mental health by facilitating regular symptom monitoring, and client-clinician feedback [17].

The multidimensional assessments collected demographics, mental health (eg, depressive, anxiety, mania, psychosis, and eating disorder symptoms), suicidal thoughts and behaviors, social and occupational functioning (eg, education, employment, and social connectedness), and substance use information of participants. A detailed description of the assessments is provided in Multimedia Appendix 1.

### Measures for Suicidal Thoughts and Behaviors

Suicidal ideation and behaviors were assessed using 2 self-reported measures. Suicidal ideation over the past month was measured using the Suicidal Ideation Attributes Scale (SIDAS, [18]). This scale is comprised of 5 items, assessing the frequency of suicidal thoughts, the individual’s ability to control these thoughts and closeness of attempting a suicide, related distress, and the extent of their impact on performing daily tasks. Each item was rated using a 10-point Likert scale where a higher value indicated more severe suicidal ideation. The SIDAS has been validated in an online survey of community-based Australian adults [18]. In addition, the Columbia-Suicide Severity Rating Scale (C-SSRS, [19]) examined suicide intent and planning over the past month and the history of suicidal attempts in the lifetime. The internal validity and consistency of C-SSRS have been validated in a multisite study with adolescents and adults [19].

### Digital Suicidality Notification System

**Figure 1:**
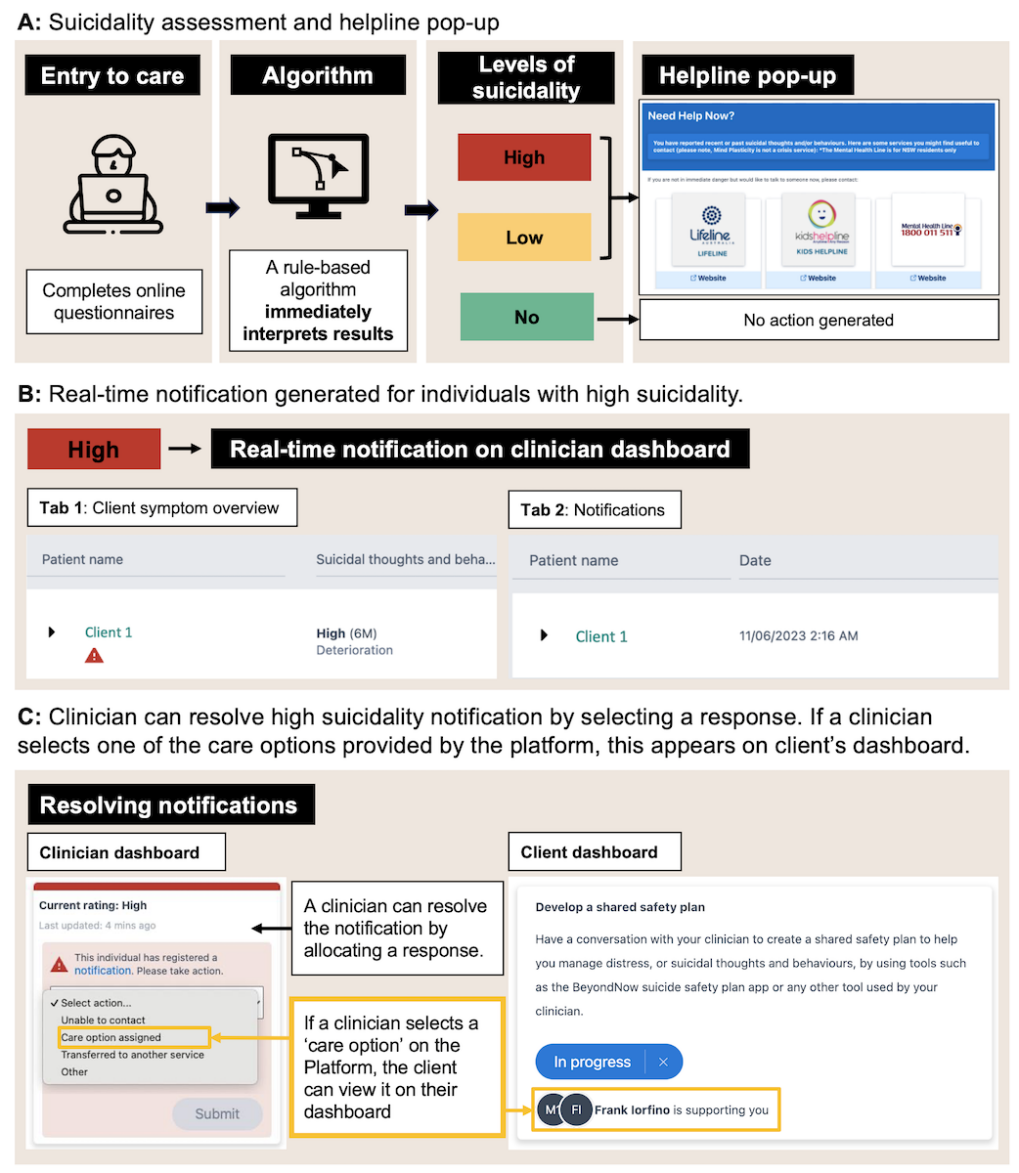
Workflow of the digital suicidality notification system.

The digital suicidality notification system is a configurable system embedded in the Innowell platform. When a young person completes the SIDAS and C-SSRS assessments, the system categorizes them into no, low, and high suicidality groups based on their results (Figure 1A). Then the system triggers appropriate actions based on their suicidality group. Thresholds for different levels of suicidality were developed by expert psychiatrists (IBH and EMS) and adjusted to existing suicidal risk management policies at services when necessary (development and thresholds of suicidality levels are provided in Multimedia Appendix 2).

After categorization, participants in low or high suicidality groups immediately receive a pop-up message with contact information for 24-hour crisis support services, such as Lifeline, Kids Helpline, and Mental Health Helpline. For individuals in the high suicidality group, the system also sends an automatic notification to treating clinicians, prompting immediate clinical actions. This notification appears as a red triangle next to the person’s name on the clinicians’ Innowell dashboard (Figure 1B).

Clinicians can resolve the notification by logging clinical actions taken in response to the suicidality detected. There are 4 available options, which are “assigned a care option,” “transferred to another service,” “unable to contact,” or “other.” Under “assigned a care option,” clinicians could further specify their clinical action by selecting one of the options provided on Innowell: “safety plan,” “safety check,” “psychological intervention,” “scheduling a new appointment,” or “other.” Resolving the notification removes the red triangle (Figure 1C).

It was not mandatory for services to resolve these notifications. Instead, the research team recommended managing notifications according to the services’ own safety and governance protocol. This flexible adaptation approach was taken for smooth integration of the system into existing clinical workflows.

Further, participants were provided with clear expectations regarding how notifications would be responded to. During onboarding, they were informed about how the data collected would be used by services, and the international standard data privacy and security measures in place to ensure data safety. Staff communicated that these services did not provide 24-hour crisis support and that the platform would only be monitored during clinical hours (eg, Monday to Friday 8 AM to 5 PM). However, automated pop-up messages provided information on external crisis resources at all times.

### Statistical Analysis

All statistical analyses were performed using R (version 4.3.1; R Core Team). In total, 3 pairwise comparisons were conducted between the suicidality groups: (1) no versus low suicidality, (2) low versus high suicidality, and (3) no versus high suicidality. Chi-square tests were used for categorical variables and Kruskal-Wallis tests were used for continuous variables. Post hoc pairwise comparisons were performed using Dunn tests, with a Bonferroni correction applied to address the 3 comparisons made for each variable and to counteract the family-wise error rate (*P*<.02). Analyses were performed with complete observations for each variable and missing values were regarded as missing at random.

In addition, an opinion statement was provided by coauthors, who are service staff from the participating services, to reflect on their experiences of using the digital notification system in youth mental health services.

### Ethical Consideration

The Northern Sydney Local Health District Human Research Ethics Committees approved this study (HREC/17/HAWKE/480), and all participants gave online informed consent (through an opt-out process). In addition, to ensure confidentiality and privacy of participant information, all data stored in the research database were deidentified and could only be accessed by researchers in this study for research purposes.

## Results

### Sample Characteristics

A total of 2021 young people completed the intake multidimensional assessment, and 226 (11%) individuals triggered a high suicidality notification. The mean age of the cohort was 20.2 (SD 2.6) years and 72% (1463/2021) were female. Approximately a quarter of participants were receiving government benefits (26%, 523/2021), and two-thirds reported that they could not independently support themselves (67%, 1361/2021). One-tenth of individuals were not in employment, education, or training (NEET; 11%, 211/1902). On average, participants reported that they were out of work for 7.4 days (SD 7.9) out of the past 30 days. Further information on demographic, clinical, social, and functional characteristics of participants has been provided in Table 1.

**Table 1:** Baseline characteristics of participants and comparisons between different levels of suicidality.

		No suicidality	Low suicidality	High suicidality	No versus low, *P* value		Low versus high, *P* value	No versus high, *P* value	Missing values, n		
Participants, n (%)		1002 (50)	793 (39)	226 (11)	—^a^		—	—	—		
Mean age (years), (SD)		20 (2.5)	20.5 (2.6)	19.8 (2.6)	<.001		<.001	—	—		
Females, n (%)		710 (71)	593 (75)	160 (71)	—		—	—	—		
With disability, n (%)		50 (5)	86 (11)	25 (11)	<.001		—	.01	—		
English speaking, n (%)		925 (92)	738 (93)	200 (89)	—		—	—	—		
Indigenous, n (%)		51 (5)	34 (4)	11 (5)	—		—	—	—		
Living alone, n (%)		62 (6)	74 (9)	23 (10)	—		—	—	—		
Single, n (%)		613 (61)	497 (63)	152 (67)	—		—	—	—		
**Education, n (%)**											8
	Secondary	720 (72)	531 (67)	160 (71)	—		—	—	—		
	Tertiary	275 (27)	261 (33)	66 (29)	—		—	—	—		
**Social and occupational function**											
	NEET^b^, n (%)	117 (12)	65 (8)	29 (13)	—		—	—	119		
	Days out of role in last 30 days, mean (SD)	6.7 (7.8)	7.3 (7.6)	10.5 (8.9)	—		<.001	<.001	—		
	Receiving government benefit, n (%)	261 (26)	202 (26)	60 (27)	—		—	—	—		
	Dependent support level, n (%)	663 (66)	536 (68)	162 (72)	—		—	—	—		
	Everyday functioning (WSAS)^c^, mean (SD)	16.9 (8.5)	19.2 (8.3)	24.2 (8.0)	<.001		<.001	<.001	81		
	Social connectedness (SSSS)^d^, mean (SD)	7.1 (3.4)	7.2 (3.3)	8.7 (3.8)	—		<.001	<.001	254		
**Personal mental health history**											
	Any family mental health history, n (%)	550 (55)	471 (59)	128 (57)	—		—	—	441		
	Mental illness history, n (%)	511 (51)	566 (71)	185 (82)	<.001		—	<.001	295		
	Mental health-related hospitalization history, n (%)	64 (6)	132 (17)	73 (32)	<.001		<.001	<.001	295		
	Sought prior treatment, n (%)	542 (54)	570 (72)	170 (75)	<.001		—	.001	293		
	Age when first sought help (y), mean (SD)	15.6 (3.6)	15.4 (3.7)	14.8 (3.9)	—		—	—	710		
	Experienced traumatic event, n (%)	350 (35)	333 (42)	107 (47)	—		—	—	388		
**Physical comorbidity, n (%)**											
	Major physical illness	255 (25)	232 (29)	64 (28)	—		—	—	388		
**Clinical presentation, mean (SD)**											
	Psychological distress (K-10)^e^	29.9 (8.7)	31.6 (8.4)	36.1 (6.8)	<.001		<.001	<.001	99		
	Depression (QIDS)^f^	12.6 (5.2)	14 (4.8)	17.7 (4.5)	<.001		<.001	<.001	304		
	Anxiety (OASIS)^g^	8.8 (4.2)	9.5 (4.1)	11.5 (4.5)	<.001		<.001	<.001	299		
	Psychosis (PQ-16)^h^	4.6 (3.8)	5 (3.5)	6.6 (4.1)	.003		<.001	<.001	381		
	Mania (ASRM)^i^	2.9 (2.9)	3 (2.9)	2.7 (2.7)	—		—	—	374		
	Eating disorder (EDE-Q)^j^	4.5 (3.0)	4.8 (3)	5.5 (3.0)	—		.001	<.001	349		
**At-risk mental states, n (%)**											
	Mania-like experience	169 (17)	178 (22)	47 (21)	<.001		—	<.001	374		
	Psychosis-like experience	269 (27)	270 (34)	100 (44)	<.001		—	<.001	—		
	Circadian disturbance	382 (38)	359 (45)	127 (56)	.003		.005	<.001	349		
**Suicidal thoughts and behaviors**											
	Self-harm history, n (%)	341 (34)	465 (59)	159 (70)	<.001		.002	<.001	346		
	Suicidal ideation (SIDAS)^k^, mean (SD)	5.1 (9.5)	9.3 (9)	26 (12.4)	<.001		<.001	<.001	179		
	Prior suicide attempt, n (%)	192 (19)	369 (47)	179 (79)	<.001		<.001	<.001	216		
**Alcohol and substance misuse, mean (SD)**											
	Alcohol (AUDIT-C)^l^	4.4 (2.3)	4.6 (2.5)	5.2 (2.6)	—		—	.005	699		
	Cannabis (ASSIST)^m^	1.5 (2.8)	1.8 (3.1)	2.3 (3.4)	—		.007	<.001	354		
	Tobacco (ASSIST)	1.3 (2.1)	1.7 (2.3)	2.2 (2.4)	.003		<.001	<.001	352		

^a^Not applicable.

^b^NEET: not in education, employment, or training.

^c^WSAS: Work and Social Adjustment Scale.

^d^SSSS: Schuster’s Social Support Scale.

^e^K-10: Kessler-10.

^f^QIDS: Quick Inventory of Depressive Symptomatology.

^g^OASIS: Overall Anxiety Severity and Impairment Scale.

^h^PQ-16: Prodromal Questionnaire.

^i^ASRM: Altman Self-Rating Mania Scale.

^j^EDE-Q: Eating Behaviors and Body Image Questionnaire

^k^SIDAS: Suicidal Ideation Attributes Scale.

^l^AUDIT-C: Alcohol Use Disorders Identification Test.

^m^ASSIST: Alcohol, Smoking and Substance Involvement Screening.

### Real-Time Helpline Pop-Up, Notification, and Response Characteristics

**Figure 2:**
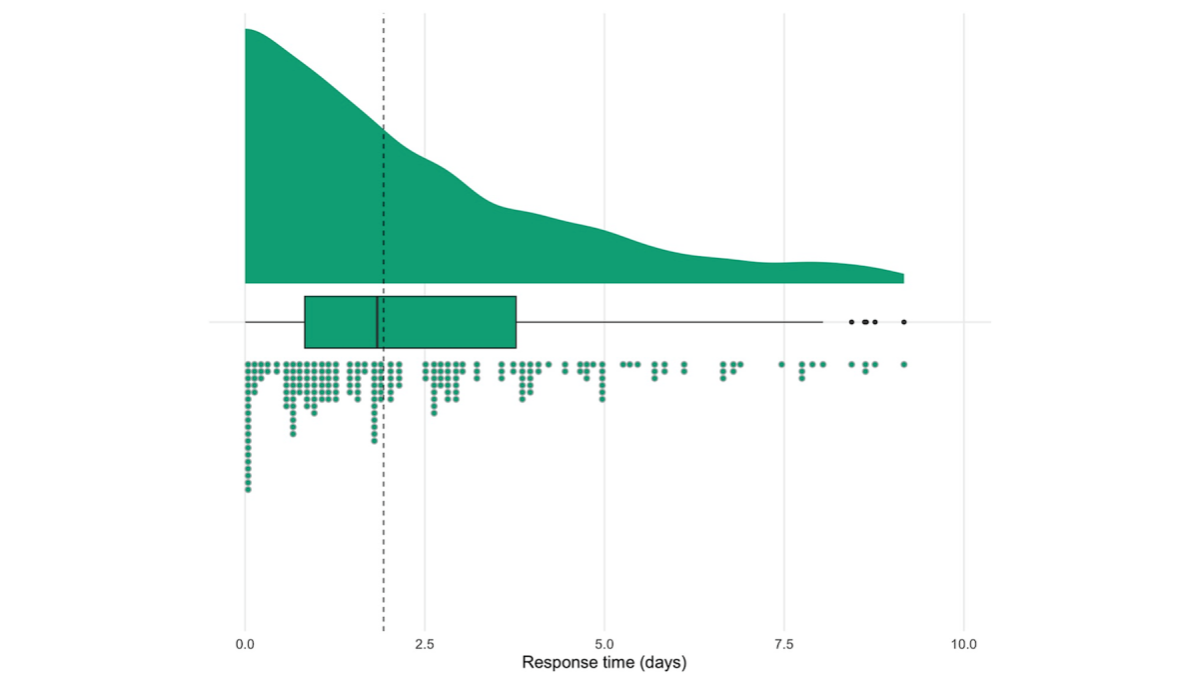
Distribution of time taken to resolve high suicidality notifications. The dashed line indicates the median response time (median 1.9 days, IQR 0-50.8 days). Outliers have been excluded in this graph for readability.

A total of 1828 helpline pop-up messages were generated, and 16% (292/1828) were accompanied by a real-time high suicidality notification. Out of the 292 notifications generated, 222 (76%) were resolved, and the median response time was 1.9 (range 0-50.8, mean 3.5, SD 5.4) days (Figure 2). Out of the 226 individuals who triggered a notification, 36 participants generated multiple notifications (mean 2.8, SD 1.4, range=2-6).

Most notifications were resolved by building a safety plan (134/222, 60%), followed by performing a safety check (39/222, 18%), initiating psychological therapies (17/222, 8%), transferring to another service (8/222, 4%; eg, general practitioner), and scheduling a new appointment with a mental health professional (4/222, 2%). Services were unable to contact 9% of individuals (20/222, Table 2).

**Table 2:** Clinical responses initiated to resolve high suicidality notifications.

Clinical response	Values, n (%)
Safety plan	134 (60)
Safety check	39 (18)
Unable to contact	20 (9)
Psychological therapy	17 (8)
Transferred to another service	8 (4)
New appointment offered	4 (2)

### Differences in Clinical and Functional Characteristics Between Suicidality Groups

Pairwise comparisons were conducted between no, low, and high suicidality groups (Table 1). The low suicidality group was older than the high suicidality group (*z* score=3.5; *P*<.001) and no suicidality group (*z* score=4, *P*<.001). Further, disability was more prevalent in the high (*χ*^2^_1_=10.8; *P*=.001) and low (*χ*^2^_1_=20.8; *P*<.001) groups compared to the no suicidality group.

Several significant differences were observed between different suicidality groups in the social and occupational functioning domains. While there were no significant differences in the NEET status between the suicidality groups, the high suicidality group spent more days out of role compared with low (*z* score=5; *P*<.001) and no suicidality groups (*z* score=6.5, *P*<.001) within the last 30 days. Similarly, the high suicidality group experienced more social disconnection compared with the low (*z* score=5.0; *P*<.001) and no suicidality groups (*z* score=5.5; *P*<.001). Also, everyday functioning was most impaired in the high suicidality group (no vs high: *z* score=11.1, *P*<.001; low vs high: *z* score=7.3, *P*<.001; no vs low: *z* score=5.6, *P*<.001).

History of mental illness was least frequently reported in the no suicidality group (no vs low: *χ*^2^_1_=48.1, *P*<.001; no vs high: *χ*^2^_1_=38.4, *P*<.001), as well as mental health-related hospitalization history (no vs low: *χ*^2^_1_=37, *P*<.001; no vs high: *χ*^2^_1_=96.1, *P*<.001; low vs high: *χ*^2^_1_=21.5, *P*<.001), and previous help-seeking history (no vs low: *χ*^2^_1_=33.9, *P*<.001; no vs high: *χ*^2^_1_=10.7, *P*<.001) compared with 2 other groups. The severity of psychological distress, depressive, and anxiety symptoms increased in the order of no, low to high suicidality groups. However, only the high suicidality group had higher levels of eating disorder behaviors (no vs high: *z* score=4.5, *P*<.001; low vs high: *z* score=3.2, *P*<.001).

The high suicidality group had the highest suicidal ideation compared with low (*z* score=13; *P*<.001) and no suicidality groups (*z* score=22.5; *P*<.001), more frequent history of self-harm (no vs high: *χ*^2^_1_=85.7, *P*<.001; low vs high: *χ*^2^_1_=9.5, *P*=.002) and higher rates of previous suicide attempts (no vs high: *χ*^2^_1_=251, *P*<.001; low vs high: *χ*^2^_1_=65.1, *P*<.001).

### Reflections on the Digital Notification System in Youth Mental Health Services

Two service managers at participating centers highlighted the strengths of using the digital notification system to manage suicidality in youth mental health services (Textbox 1). The key strengths identified were service triage and wait time management, client engagement, facilitation of online resource use, and clinical decision-making support.

**Strengths of the digital notification system – insights from clinical practice.**

Service triage and wait time management

- The system supports service triage by reducing wait times for young people with high suicidality, ensuring they receive care at the time of their need. The support of a digital system is especially helpful during periods of low staffing for maintaining the quality of care and responsiveness.

Client engagement

- Reviewing assessment results from the digital platform (Innowell) can start conversations for young people who might have not discussed their suicidality previously. However, achieving this requires a service-wide adoption of the digital technology. All service staff and clinicians need to understand and consistently use the platform throughout the client journey to maximize its potential as an engagement and communication tool.

Facilitation of online resource use

- Many young people are willing to engage in safety planning on the platform when guided by a clinician. This interaction not only helps to manage suicidality but also builds rapport and familiarizes young people with using the platform effectively.

Clinical decision-making support

- The care options provided on the digital platform offer guidance to clinicians, particularly those with limited experience to build confidence in addressing suicidality in young people.

## Discussion

### Principal Results

This study demonstrates that a digital suicidality notification system successfully initiated subsequent clinical responses for young people who expressed high suicidal ideation and behaviors. Of the high suicidality notifications, 76% (222/292) were resolved, and the median response time was 1.9 days (range 0-50.8 days). Building on its initial evaluation in 2017 [11], the high rate of resolved notifications over a 5-year period suggests that the system was accepted and used in real-world clinical settings.

Further, the study reveals the multidimensional and complex symptom presentations of young people with low or high suicidality levels. Compared with the no suicidality group, the low and high groups exhibited higher levels of clinical symptoms, more severe social and occupational impairments, and circadian disturbances. A history of mental health diagnosis and related hospitalization were more frequently reported in these groups.

Together, this study demonstrates the effectiveness of the digital notification system to detect suicidal needs, reveal associated symptom complexity, and trigger appropriate responses in youth mental health services.

### Digital Systems for Rapid and Personalized Management of Suicidality

This evaluation demonstrated the potential of the digital notification system to facilitate stratification and personalization of care. The results show that most young people who elicited a notification received evidence-based treatment, including brief interventions (ie, safety checks and safety plan [20-23]), and long-term psychological interventions [24-26]. Alternatively, the system facilitated care stratification by expediting existing appointments and initiating care coordination, such as service transfers. Such cascades of clinical actions would have been delayed without this system. Research indicates that reducing wait times for individuals with high levels of suicidality could be critical due to its strong associations with severe mental illnesses [27], the risk of recurrence of suicidal behaviors [28], and comorbidity [29]. Further, there is a high likelihood that individuals present to health services before suicide [30]. Therefore, expedited clinical responses facilitated by the system may help reduce suicide rates through early detection and proactive care [5,31]. Addressing broader clinical and psychosocial needs associated with suicidal thoughts and behaviors could mitigate severe outcomes, such as suicide attempts [32]. and emergency department presentations [33].

While the tool led to appropriate clinical actions, a 2-day median response rate may raise concerns about its timeliness for supporting youth who are highly suicidal. However, it is important to recognize that these participating services did not provide 24-hour crisis support, and young people were informed about the system’s limited monitoring hours. A potential future use of this system could include automatic transfer of notification information to 24-hour crisis support services outside of service operating hours, to help young people receive immediate help.

Another research direction of this notification system is its potential to be reconfigured to detect different combinations of symptoms that indicate the need for rapid intervention, including psychosis-like symptoms and severe social impairments. In addition, the system could be enhanced by embedding machine learning tools to detect intraindividual changes in symptom trajectories [34,35], screen complex symptom presentations [36], or analyze linguistic patterns to detect suicidality based on user-created content [37,38]. Integrating these tools into the notification system could facilitate services to quickly understand young people’s needs and take subsequent actions. This can be done by either providing direct care or coordinating with services that can deliver the required treatments.

### Multidimensional Needs of Youth With High Suicidality

Notably, this study highlights the concurrent multidimensional impairments found in individuals with high levels of suicidality. Those who triggered a notification exhibited greater clinical, social, and functional impairments compared to groups with lower suicidality. These findings align with previous suicide research, where mental illnesses [39], psychosis-like experiences [40], irregular sleep-wake cycles [41], and eating disorders [42] have been associated with an increased likelihood of a suicide attempt. In particular, the association between high suicidality and severe social and occupational impairments, such as more days out of work and greater social disconnection, supports the causal relationship between these factors previously proposed [43-46].

These findings emphasize the importance of multidimensional assessments in addressing suicidality. Considering the recent critique of the marginally positive effect sizes of common suicide prevention interventions [25,47], deploying multidimensional measures could improve the identification of individuals’ underlying mechanisms and support the formulation of personalized treatments. Digital platforms offer several advantages in this context, offering increased accessibility, privacy for clients to complete assessments, and reduced time and labor demand for services [48,49].

### Implications in Youth Mental Health Services

A successful integration of the notification system into service workflow is crucial to maximize its benefits, as outlined in Textbox 1. Key strategies to facilitate implementation are the integration of the digital platform into clinical governance and the designation of key individuals to monitor notifications.

Integration of the digital system into clinical governance involves establishing organizational protocols that explicitly outline its use within the existing workflow. These protocols should be codesigned with clinicians and service staff and regularly reviewed to ensure acceptability and feasibility. Training sessions and modeling its use by senior clinicians can promote implementation by emphasizing its clinical utility [50,51]. Further, addressing common barriers such as data privacy concerns through transparent communication of data confidentiality would be critical for building trust in the system [52].

In addition, designating service staff to monitor and support digital platform use can facilitate the escalation of notifications into clinical actions. The concept of a digital navigator is one such example [53,54]. A digital navigator in services can alleviate the additional burden placed on clinicians by constantly monitoring the notification system and ensuring timely escalation of notifications. They can also be trained to deliver brief interventions, such as safety planning and safety checks, to help manage service demands [50,55].

### Limitations

There are several limitations that should be taken into consideration. First, a higher proportion of participants in this study reported a history of self-harm or suicide attempts compared with previous studies [56,57]. This characteristic might influence the types of notifications generated and the subsequent responses. However, the primary objective of this study was to demonstrate the feasibility and acceptability of a digital notification system within a mental health service setting. Therefore, the system’s high response rate, despite the frequent generation of alerts, strongly suggests its potential utility in managing cases with high clinical needs. Further research is required to assess its generalizability to populations with different characteristics and in various health care settings. Second, some may suggest that the threshold for a high suicidality notification is too sensitive, capturing individuals who may not have acute needs. However, the primary aim of the notification system is not to identify individuals with immediate suicidal risk. Rather, the notification is designed to inform clinicians about an individual's needs that require response within a reasonable time frame, due to the long-term implications for mental health. This notification system is a tool to facilitate personalized care. A part of enhancing tailored care could involve the use of an advanced machine learning tool that provides a more comprehensive understanding of the volatility of one’s suicidal ideation and behaviors using their longitudinal data [34,58]. Third, the self-reported measures used can be subject to bias or underreporting. While the self-reported data should be supplemented with clinician reports, the measures used have been well-validated for detecting suicidality. Further, when data is collected longitudinally, it becomes a powerful tool for detecting intraindividual changes [59]. Finally, this study does not assess the impact of this notification system on future illness trajectories of youth. Future studies should evaluate the long-term effectiveness of the interventions facilitated by notifications, individual variability in treatment response, and its impact on overall suicidality expressed among youth.

### Conclusions

This study demonstrates the effectiveness of a digital suicidality notification system in initiating timely clinical actions for young people expressing high suicidality. Further, it highlights that using multidimensional assessments for screening and monitoring of symptoms can be critical for providing personalized treatments. Future research should focus on exploring client and clinician perspectives on using the system to improve its implementation. In addition, examining the longitudinal outcomes of clinical actions initiated by the system would provide insights into their impact on mental health trajectories and suicide rates. Ultimately, this study demonstrates the system’s capacity to enhance service responses to suicidality detected in youth through efficient screening, continuous monitoring, and personalized interventions.

## Appendix

Multimedia Appendix 1 A complete list of measures used in the Innowell Platform.

Multimedia Appendix 2 Thresholds for suicidality stratification.

## Abbreviations

C-SSRS: Columbia-Suicide Severity Rating Scale
NEET: Not in employment, education, or training
SIDAS: Suicidal Ideation Attributes Scale
