# Supplementary material for: A Digital Approach for Addressing Suicidal Ideation and Behaviors in Youth Mental Health Services: Observational Study
Source: J Med Internet Res. 2024 Dec 18;26:e60879. doi: 10.2196/60879 (PMC11694056; doi:10.2196/60879)
Supplement: Multimedia Appendix 1 [file jmir_v26i1e60879_app1.docx]

**Multimedia Appendix 1**: A complete list of measures used in the Innowell platform.

**Demography**

Participants’ age, sex, level of education, presence of disability, indigenous background, and relationship status were collected.

**Mental Health**

Participants’ previous mental health illnesses, hospitalisation history, and treatment utilisation were recorded. Additionally, history of help-seeking, personal and family mental illness were collected.

Various measures were used to identify participants’ current mental health symptom presentation. The Kessler Psychological Distress Scale (K10, [1]) and Overall Anxiety Severity and Impairment Scale (OASIS, [2)) were used to measure participants' psychological distress and anxiety, respectively. The Quick Inventory of Depressive Symptomatology – Self-Report, (QIDS, [3]) was used to assess depressive symptoms, the Altman Self-Rating Mania Scale (ASRM, [4]) for mania-like experiences, the Prodromal Questionnaire (PQ-16, [5]) for psychosis-like experiences, and the PTSD-5, [6] for post-traumatic experiences.

**Social and occupational functioning**

The individuals’ status of not in education, employment, and training (NEET) was collected. If the individual identified themselves as being in a NEET status, the number of days that they were unable to carry out their usual activities in the last 30 days were recorded. Further, everyday functioning (The Work and Social Adjustment Scale WSAS, [7]) and social connectedness (Schuster’s Social Support Scale, SSSS, [8]) were assessed. Participants’ government benefit status and their ability to independently support themselves was acquired.

**Self-harm**

An adapted version of the Brief Non-Suicidal Self-Injury Assessment Tool, [9] was used for history, recency and future intention of self-harm.

**Alcohol and Substance Use**

Participants’ use of alcohol, tobacco and other drug use was assessed using a combination of 3 measures. The Alcohol Use Disorders Identification Test (AUDIT-C, [10]) was used to assess the frequency of use, Alcohol, Smoking, and Substance Involvement Screening Test (ASSIST, [11]), assessed the health, social, legal, and financial impacts that resulted from substance use.

**Eating disorder**

An adapted version of the Eating Disorders Examination (EDE-Q, [12]) assessed participants’ eating behaviours such as binge eating, purging, strict dieting, and body image importance.

**Circadian rhythm disturbance**

The Pittsburgh Sleep Quality Index [13] and the Munich Chronotype Questionnaire [14] were used to collect sleep and wake time, sleep duration, and quality of sleep. Also, a question on restorative sleep was added, given its significance demonstrated in the past literature [15].

**References**

1. Kessler RC, Andrews G, Colpe LJ, Hiripi E, Mroczek DK, Normand SL, et al. Short screening scales to monitor population prevalences and trends in non-specific psychological distress. Psychol Med. 2002;32(6):959-76.

2. Moore SA, Welch SS, Michonski J, Poquiz J, Osborne TL, Sayrs J, et al. Psychometric evaluation of the Overall Anxiety Severity And Impairment Scale (OASIS) in individuals seeking outpatient specialty treatment for anxiety-related disorders. J Affect Disord. 2015;175:463-70.

3. Rush AJ, Trivedi MH, Ibrahim HM, Carmody TJ, Arnow B, Klein DN, et al. The 16-Item Quick Inventory of Depressive Symptomatology (QIDS), clinician rating (QIDS-C), and self-report (QIDS-SR): a psychometric evaluation in patients with chronic major depression. Biol Psychiatry. 2003;54(5):573-83.

4. Altman EG, Hedeker D, Peterson JL, Davis JM. The Altman Self-Rating Mania Scale. Biol Psychiatry. 1997;42(10):948-55.

5. Ising HK, Veling W, Loewy RL, Rietveld MW, Rietdijk J, Dragt S, et al. The validity of the 16-item version of the Prodromal Questionnaire (PQ-16) to screen for ultra high risk of developing psychosis in the general help-seeking population. Schizophr Bull. 2012;38(6):1288-96.

6. Prins A, Bovin MJ, Smolenski DJ, Marx BP, Kimerling R, Jenkins-Guarnieri MA, et al. The Primary Care PTSD Screen for DSM-5 (PC-PTSD-5): Development and Evaluation Within a Veteran Primary Care Sample. J Gen Intern Med. 2016;31(10):1206-11.

7. Mundt JC, Marks IM, Shear MK, Greist JH. The Work and Social Adjustment Scale: a simple measure of impairment in functioning. Br J Psychiatry. 2002;180:461-4.

8. Schuster TL, Kessler RC, Aseltine Jr. RH. Supportive interactions, negative interactions, and depressed mood. American Journal of Community Psychology. 1990;18(3):423-38.

9. Whitlock J, Exner-Cortens D, Purington A. Assessment of nonsuicidal self-injury: Development and initial validation of the Non-Suicidal Self-Injury–Assessment Tool (NSSI-AT). Psychological Assessment. 2014;26:935-46.

10. Bush K, Kivlahan DR, McDonell MB, Fihn SD, Bradley KA, Project ACQI. The AUDIT alcohol consumption questions (AUDIT-C): an effective brief screening test for problem drinking. Archives of internal medicine. 1998;158(16):1789-95.

11. WHO ASSIST Working Group. The alcohol, smoking and substance involvement screening test (ASSIST): development, reliability and feasibility. Addiction. 2002;97(9):1183-94.

12. Hay PJ, Mond J, Buttner P, Darby A. Eating disorder behaviors are increasing: findings from two sequential community surveys in South Australia. PLoS One. 2008;3(2):e1541.

13. Buysse DJ, Reynolds CF, 3rd, Monk TH, Berman SR, Kupfer DJ. The Pittsburgh Sleep Quality Index: a new instrument for psychiatric practice and research. Psychiatry Res. 1989;28(2):193-213.

14. Roenneberg T, Wirz-Justice A, Merrow M. Life between Clocks: Daily Temporal Patterns of Human Chronotypes. Journal of Biological Rhythms. 2003;18(1):80-90.

15. Vernon MK, Dugar A, Revicki D, Treglia M, Buysse D. Measurement of non-restorative sleep in insomnia: A review of the literature. Sleep Med Rev. 2010;14(3):205-12.
