# Supplementary material for: A Digital Approach for Addressing Suicidal Ideation and Behaviors in Youth Mental Health Services: Observational Study
Source: J Med Internet Res. 2024 Dec 18;26:e60879. doi: 10.2196/60879 (PMC11694056; doi:10.2196/60879)
Supplement: Multimedia Appendix 2 [file jmir_v26i1e60879_app2.docx]

**Multimedia Appendix 2:**

Table 1 provides detail of suicidality categorization thresholds. These criteria were determined by two expert psychiatrists (IBH and EMS) and guided by service policies.

A high suicidality was determined by a cut-off score of 21 from the Suicidal Ideation Attributes Scale (SIDAS, [1]). While any ideation could indicate risk for suicidal behaviors, scores ≥21 had 95.8% specificity for presence of a suicide plan in the past year and 94.9% specificity for presence of preparation/attempt in the past year. Furthermore, a meta-analysis showed that intent to act on suicidal ideation, and previous history of suicide attempt were predictive of future suicidal behaviors[2]. Hence, high suicidal ideation and previous history of suicide attempts were used to indicate high risk for suicidal behaviors.

When the Innowell platform was implemented in services, the thresholds were reviewed again by service leadership to ensure that they aligned with respective services’ risk escalation policies.

**Table 1**: Thresholds for suicidality stratification

| None | No suicide ideation, intent or plans in the past month.  AND  No reported history of suicidal behaviour |
| --- | --- |
| Low | Low suicidal ideation (Suicidal Ideation Attributes Scale ≤21) and NO plan or intent in past month  OR  High suicidal ideation (Suicidal Ideation Attributes Scale ≥21), and no plan or intent in past month  OR  Suicidal attempt more than 3 months ago |
| High | Suicidal ideation with intent in the past month  OR  Suicidal ideation with intent and plans in the past month  OR  Suicide attempt within past 3 months  OR  Suicide attempt in the past month |

Reference:

1. van Spijker, B.A., et al., *The suicidal ideation attributes scale (SIDAS): Community-based validation study of a new scale for the measurement of suicidal ideation.* Suicide Life Threat Behav, 2014. **44**(4): p. 408-19.

2. Greist, J.H., et al., *Predictive Value of Baseline Electronic Columbia-Suicide Severity Rating Scale (eC-SSRS) Assessments for Identifying Risk of Prospective Reports of Suicidal Behavior During Research Participation.* Innov Clin Neurosci, 2014. **11**(9-10): p. 23-31.
